# Impact of Circulating Triglycerides Concentration on Atherosclerotic Disease Status in Middle-Aged Saudi Arabian Dwellers

**DOI:** 10.3390/nu10111642

**Published:** 2018-11-02

**Authors:** Wedad Azhar, Bartek Buczkowski, Christopher Smith, Gladys Onambele-Pearson

**Affiliations:** 1 Department of Health Professions, Faculty of Health, Psychology and Social Care, Manchester Metropolitan University, Manchester M15 6BG, UK; WEDAD-FOUAD.O.AZHAR@stu.mmu.ac.uk (W.A.); B.Buczkowski@mmu.ac.uk (B.B.); C.X.Smith@mmu.ac.uk (C.S.); 2 Department of Exercise & Sport Science, Manchester Metropolitan University, Crewe, Cheshire CW1 5DU, UK

**Keywords:** triglycerides, atherosclerosis, cardiometabolic health, central blood pressure, peripheral blood pressure, composite z-score

## Abstract

A number of food micronutrients are reported to influence markers of cardio-metabolic health. There is an expectation that there may be an optimal endocrine profile, with triglycerides as a key factor, which may help minimise atherosclerotic disease and associated risk factors. This study involved 84 participants aged (mean ± SD) 48.2 ± 8.3 years from both sexes segregated into *n* = 30 controls, *n* = 25 at-risk, and *n* = 29 diagnosed with atherosclerosis, including 20 participants using statins. Atherosclerosis status and risk factors were assessed using a combination of clinical records, C-reactive protein (CRP), blood glucose (FBG), lipids profiles, vascular structural and functional characteristics (including carotid-radial pulse wave velocity (CR-PWV), central systolic blood pressure (C-SBP), peripheral systolic blood pressure (P-SBP), peripheral diastolic blood pressure (P-DBP), carotid intima-media thickness (IMT), and carotid artery inter-adventitial diameter (IAD)). There was a significant difference in triglycerides (TG) levels between the clinical groups (*p* < 0.05) and between the users and non-users of statin (*p* < 0.001). Significant associations were distinguished between TG and CRP, FBG, high-density lipoprotein (HDL), C-SBP, P-SBP, P-DBP, CR-PWV, heart rate (HR), and body weight in the pooled sample (*p* < 0.05). In non-users of statin, TG was associated with C-SBP, P-SBP, P-DBP, and HR. In sub-clinical groups, TG was also associated with most of the blood markers. After controlling for statin use, composite z-score analysis revealed 48%, 2%, and 0% differences in in vivo vascular phenotype between high and low TG subgroups in controls, at-risk, and diagnosed atherosclerosis groups, respectively. Thus, TG levels seem to be good indicators for incidence and risk factors of atherosclerosis.

## 1. Introduction

Atherosclerosis is the most common type of heart disease and a major cause of death worldwide [1]. It is a condition that thickens artery walls due to the accumulation of white blood cells and fatty substances (fatty deposits) [2]. Many risk factors such as advanced age, being a male, inheritance of premature atherosclerotic disease, hypercholesterolemia, hypertension, hyperglycemia, smoking, and obesity can increase the chance of developing atherosclerosis [3,4,5,6].

Atherosclerosis is an asymptomatic condition until patients present angina or other complications of the disease [7,8]. Risk of development of atherosclerosis can be predicted from the disease risk factors and/or inflammatory markers such as hyperlipidemia, hypertension, hyperglycemia, and high C-reactive protein (CRP) [2,9]. The assessment of the arteries’ function, resistance, and stiffness is used to diagnose the condition, which can be performed using electrocardiogram, computerised tomography (CT) scan, exercise stress tests, and ultrasound as non-invasive techniques [3,10]. The measurement of blood flow, vessel diameter, or artery stiffness, such as coronary microvascular vasoreactivity, flow-mediated dilation (FMD), or pulse wave velocity (PWV), are also innovative effective methods for diagnoses of vascular functions [11,12]. In complicated cases of the disease and for treatment purpose, digital subtraction angiography (DSA) and coronary artery bypass grafting (CABG) might be used [13,14]. To diagnose the cases, a valuable non-intensive method is the use of color Doppler ultrasonography to assess major arteries’ (e.g., carotid) intima-media thickness (IMT) and inter-adventitial diameter (IAD) [14,15].

Hyperlipidaemia indicates abnormal levels of one or more forms of plasma lipids including low-density lipoprotein (LDL) cholesterol, high-density lipoprotein (HDL) cholesterol, and triglycerides (TG) [16,17,18]. Although high LDL and low HDL are well established major risk factors of atherosclerosis, especially given the correlation of higher total levels of the HDL particles with reduced incidence of vascular disease in various clinical trials and epidemiological studies [19], the extent to which TG directly promote atherosclerosis development and incidence is unclear [20]. Optimal amounts of TG are necessary for many body functions, being a key source of energy; it can be obtained from dietary sources and is produced by the liver [21]. On the other hand, a high concentration of TG is considered to be a marker for heart disease risk factors including obesity, diabetes, hypertension, and high cholesterol levels [20]. TG are the most common type of fat in the body [21] and are mainly carried in the bloodstream by chylomicrons and very low density lipoprotein (vLDL), which gives rise to LDL particles. A high concentration of LDL may lead to endothelial dysfunction and is associated with increased risk of atherosclerosis [6,20]. Indeed, the current thoughts are that the LDL fragments lodge in arterial walls, which have been damaged where they attract white cells and platelets, hence forming plaques and developing into atherosclerosis over years. In accordance with the above, previous mechanistic work links disruptions in triglyceride metabolism (and thus subsequent atherosclerosis) to mutations in certain genes such as APOA5, APOC2, GPIHBP1, or LMF1, as well as autoimmune antibodies to lipoprotein lipase. Lipase is the enzyme that catalyses the hydrolysis of the triacylglycerol component of circulating chylomicrons and vLDL, which supplies non-esterified fatty acids and 2-monoacylglycerol for tissue utilisation [22].

This study investigates whether there is an association between atherosclerosis disease incidence and risk factors and TG. The objective was to add to the body of evidence on the link between concentrations of TG independently linked to clinical atherosclerosis status.

## 2. Methods

### 2.1. Subject Selection

A total of 84 volunteers, aged 35 to 60 years, including 14 males and 70 females, were selected to participate in the study, leading to a sex bias in our recruitment. In this study, data were collected by a female researcher. More females participated in the study because of the social mores of Saudi Arabia, which place restrictions on how men and women can interact. Participants did not have a confounding medical condition such as liver or kidney disease, metabolic bone disease (e.g., osteoporosis), cancer, or hypercortisolism. Participants had not undergone bypass surgery, and were not currently pregnant or breastfeeding.

Participants were segregated into *n* = 30 controls (CG), who had never been diagnosed with any systemic disease; *n* = 25 at-risk of developing atherosclerosis group (ARG), who had at least one of the major risk factor diseases (hypertension, diabetes, and/or hyperlipidaemia); and *n* = 29 diagnosed with atherosclerosis group (DAG). Participants were either patients at the cardiovascular clinic at Al -Noor Specialist Hospital, Saudi Arabia, or were invited to the clinic to participate in the study. Ethical approval was obtained from the Faculty Research Ethics Committee at Manchester Metropolitan University and from the Committee of Medical Ethics at Al-Noor Specialist Hospital in Saudi Arabia.

### 2.2. Study Design

After obtaining the information sheet and signing the consent form, all participants completed a demographic lifestyle questionnaire, and provided information on their medical history and usage of medication. Anthropometric measurements including height, weight, and waist and hip circumference were recorded. Fasted blood samples were handled by the laboratory at the hospital for analysis of triglycerides (TG), 25-hydroxyvitamin D (25(OH)D), C-reactive protein (CRP), blood glucose (FBG), total cholesterol (TC), high-density lipoprotein (HDL), and low-density lipoprotein (LDL). Vascular structural and functional characteristics were performed after volunteers had laid flat on the test bed for 10 min in a controlled environment (quiet, lit, and temperature set at 23 °C [23,24]). A brightness and colour Doppler ultrasound (MyLab 70, Esaote, Genoa, Italy) was used to assess carotid intima-media thickness (IMT) and carotid artery inter-adventitial diameter (IAD). A non-invasive pulse-wave velocity device (Complior, Alam Medical, Vincennes, France) and associated software (V1.9.4, Alam Medical, Vincennes, France) were used to assess pulse wave velocity (PWV) and central systolic blood pressure (CSBP). Peripheral systolic blood pressure (PSBP), peripheral diastolic blood pressure (PDBP), and heart rate (HR) were measured using an electronic sphygmomanometer (CARESCAPE V100 Monitor, GE Healthcare, St. Louis, MO, USA).

#### 2.2.1. Health Questionnaire and Anthropometry

A questionnaire was designed in-house to collect information on gender, age, atherosclerosis-relevant health information, and use (frequency and type) of medication.

Anthropometric measurements were taken and recorded by the trained principal investigator. To establish the participants’ body mass index (BMI), height in centimeters and mass in kilograms were measured using a stadiometer and an electronic scale (Doran Scales, Charles, IL, USA), respectively. The participants stood on the scale wearing light clothing without their shoes or any additional accessories. To establish the waist to hip ratio (WHR), measurements of the waist and hip circumference were taken in centimeters. The participants stood straight with feet close together, resting arms down, and relaxed abdominal muscles. Using a measuring tape, circumference of the hip was taken at the widest part of the buttocks and of the waist at the midpoint between the hip and the last rib cage bone.

#### 2.2.2. Blood Test Measurements

Fasting blood samples were collected and handled by trained phlebotomists in the hospital. Enzymatic methods were used to determine lipid profiles using the Roche enzymatic colorimetric assay system (Roche c 501, Indianapolis, IN, USA). Normal ranges are set between 30 and 200 mg/dL for TG, 50 and 200 mg/dL for TC, 35 and 55 mg/dL for HDL, and 0.01 < 150 mg/dL for LDL. For the measurement of FBG, a hexokinase enzymatic reference method using a Roche Cobas c 501 analyser (Indianapolis, IN, USA) was used. Normal concentrations are between 70 and 115 mg/dL. Finally, high sensitivity CRP measurements were determined using an in vitro diagnostic reagent (Siemens BN II/BN ProSpect^®^ system, Marburg, Germany). Normal concentrations are between 0.0001 and 0.3 mg/dL.

#### 2.2.3. Vascular Structure and Kinetic Measurements

**Figure 1:**
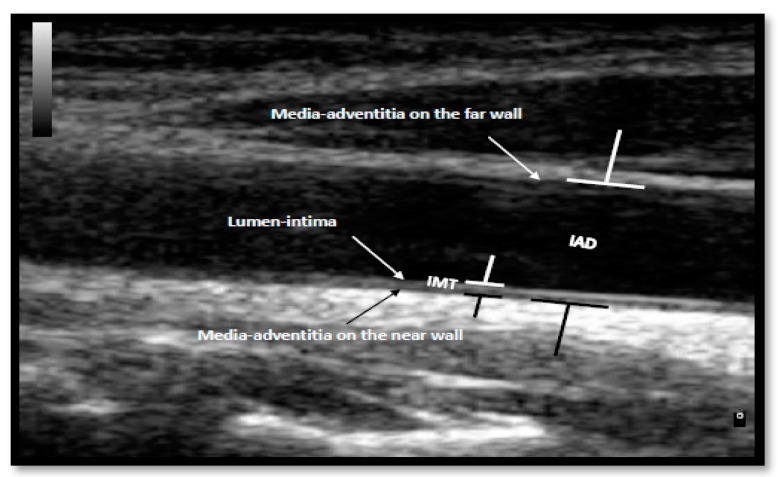
Illustration of intima-media thickness (IMT) and inter-adventitial diameter (IAD) measurements on a participant’s common carotid artery (CCA) ultrasound image.

The measurements of IMT and IAD were assessed using a brightness and colour Doppler ultrasound (MyLab 70, Esaote, Genoa, Italy). The measurements were performed on a scan image of the common carotid artery (CCA) during diastolic phase, 10.00 mm away from the carotid node. Three measurements at three different points along the vessel were taken and the mean was recorded. IMT was determined by measuring the distance between the lumen-intima interface and the medial-adventitial interface in the CCA (Figure 1). IMT is considered normal at <0.75 mm; at risk of atherosclerosis between the 0.75 mm and 1.00 mm; and atherosclerosis, as is typical, at >1.00 mm. For the IAD measurements, the distance between the medial-adventitial interfaces on the near wall and the medial-adventitial interface on the far wall on the CCA was recorded (Figure 1).

**Figure 2:**
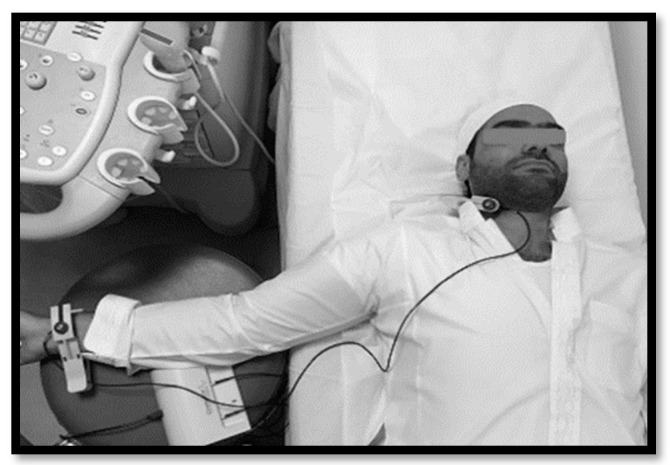
The pulse wave analysis test being performed on a participant.

CR-PWV and C-SBP were determined using a pulse-wave velocity device (Complior, Alam Medical, Vincennes, France) and software (V1.9.4, Alam Medical, Vincennes, France). The examinations were performed on the carotid and radial arteries by placing the relevant hands-free sensors on the key anatomical sites on the participant’s neck and wrist (Figure 2). The participant’s information (ID, gender, date of birth, P-SBP, P-DBP, height, mass, and distance between the carotid and radial arteries) was recorded in the system. The participants’ arms and legs were not allowed to come into contact with the rest of the body in order to ensure a clear signal was obtained. The software gives a reading after 10 valid pulses are obtained. For accurate readings, a quality score above 80% was required. The CR-PWV was calculated by the software as the distance between the two measured points divided by the transit time of the waves between those two points (m/s). The C-SBP is the pressure in the carotid artery.

### 2.3. Statistical Analyses

Statistical analysis was performed using SPSS v.24 (Inc., Chicago, IL, USA). Data were tested for normal distribution using the Kolgomorov–Smirnov test. Homogeneity of variance was assessed using the Levene’s test. For normally distributed data, one-way analysis of variance (ANOVA), with Bonferroni pairwise comparisons, was carried out post-hoc for comparison between sub-groups. Non-normally distributed data were deemed suitable for non-parametric tests and as such, Kruskal–Wallis pairwise comparisons with appropriate post-hoc Mann–Whitney tests were carried out on these. Additionally, where data were continuous rather than grouped, Spearman rho correlations were carried out. Student’s *t*-test on the Z-score transformed equation coefficients was carried out to determine any clinical group effect on the slope between TG and markers of cardiovascular health. Where the correlation in two clinical groups was not significant (between triglyceride levels and a cardiovascular outcome), their related slopes were not compared. Data are presented as mean ± standard deviation for parametric data, or median (interquartile range) for non-parametric data. Statistical significance was accepted at *p* ≤ 0.05.

## 3. Results

Eighty-four participants with complete data without outliers (outlier labelling role for interquartile range (IQR) of 3) were included in the study. General characteristics of the participants are provided in Table 1.

### 3.1. The Differences in Triglyceride Levels in the Three Clinical Groups

To investigate the difference in TG levels between the clinical groups, non-parametric tests were performed. A Kruskal–Wallis test revealed a statistically significant main effect of the clinical group on TG levels (χ^2^ (2) = 9.27, *p* = 0.01). The significant differences were between CG and ARG (U = 206, *p* = 0.004) and CG and DAG (U = 284, *p* = 0.02). Additionally, significant differences in TG concentrations were observed between statin users and non-users (*p* = 0.001) within the clinical groups. Table 2 illustrates the distribution of all of the study variables among the clinical groups. The *p* values for the difference between the clinical groups in each of the variables were obtained using the Kruskal–Wallis test.

### 3.2. Triglycerides Associations with Vascular Structure and Kinetic Data, as Well as Endocrine Profile

Based on Spearman’s correlation (one-tailed), TG was significantly correlated with CRP (R = 0.219, *p =* 0.023), FBG (R = 0.492, *p* < 0.0001), and HDL (R = −0.424, *p* < 0.0001) blood levels in the pooled sample. Further correlations were observed within the study clinical groups. In the control group, TG was significantly associated with HDL (R = −0.474, *p =* 0.004) and LDL (R = 0.427, *p =* 0.009). In the at-risk group, TG was observed to be associated with FBG (R = 0.576, *p =* 0.001), TC (R = 0.452, *p =* 0.012), HDL (R = −0.349, *p =* 0.044), and LDL (R = 0.428, *p =* 0.016). Finally, in the developing atherosclerosis group, TG was significantly correlated with FBG (R = 0.494, *p =* 0.003) and HDL (R = −0.446, *p =* 0.008).

From the vascular structure and kinetic data, TG was also associated with CR-PWV (R = 0.194, *p =* 0.039), C-SBP (R = 0.192, *p =* 0.040), P-SBP (R = 0.184, *p =* 0.047), P-DBP (R = 0.309, *p =* 0.002), and HR (R = 0.263, *p =* 0.008) in the pooled sample. No correlations between TG and vascular structure parameters exist within the subsets of clinical groups.

Given the impact of statins on blood lipid levels, as well as the noted difference in TG between statin users and non-users in pooled samples, it was pertinent that the non-pharmacologically modulated TG levels be considered in follow-up analyses. Thus, in non-users of statins, a number of significant Spearman’s correlations remained between TG and vascular markers of atherosclerosis. The correlations were between TG and C-SBP (R = 0.218, *p =* 0.042), P-DBP (R = 0.211, *p =* 0.047), P-DBP (R = 0.314, *p =* 0.006), and HR (R = 0.285, *p =* 0.011).

**Figure 3:**
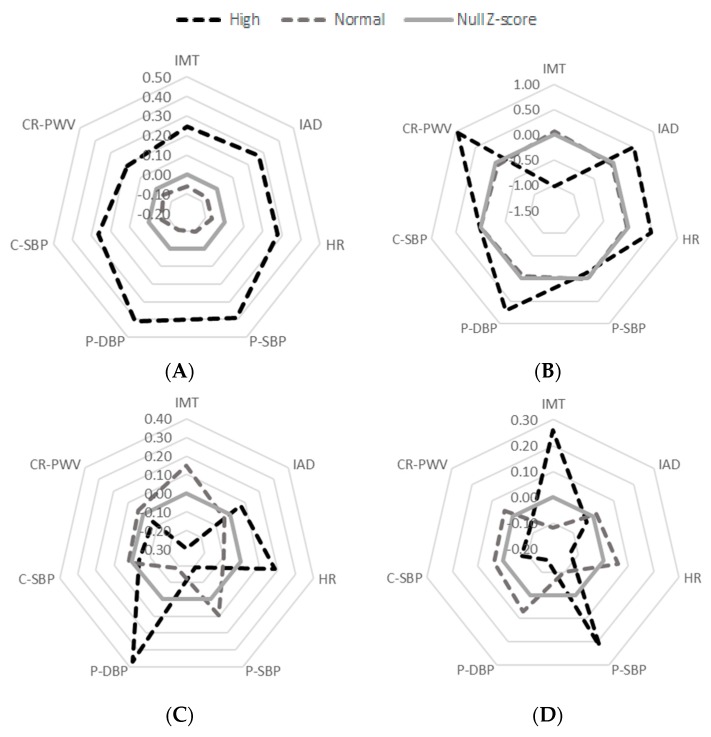
Reader graphs of normal/high TG concentrations against the vascular markers of atherosclerosis without the usage of Statin in (**A**) the total sample, (**B**) control group (CG), (**C**) at-risk of developing atherosclerosis group (ARG), and (**D**) diagnosed with atherosclerosis group (DAG). HR—heart rate; C-SBP—central systolic blood pressure; P-SBP—peripheral systolic blood pressure; P-DBP—peripheral diastolic blood pressure; CR-PWV—carotid-radial pulse wave velocity; IMT—intima-media thickness; IAD—inter-adventitial diameter.

While the information of each of the seven in vivo markers of vascular structure and function is an interesting set of data in itself when considering each clinical group, the holistic view of the entire vascular phenotype profile is arguably a more elegant and comprehensive approach to understanding how these parameters relate to one another. Z-scores were thus computed for each of the in vivo parameters to enable them to be plotted on the same scale within a single radar graph. The data were segregated in a binary fashion, comparing non-statin users still presenting a high TG profile against statin users with a normal TG profile (see Figure 3 below).

Thus, the unit weighed (and direction corrected) composite z-score analysis shows that in the pooled non-medicated sample, while the normal TG participants tend to show values 2% below the median, the high TG participants showed values 70% above the median, a 67% difference in in vivo vascular phenotype, with all seven parameters being greater in the high TG subgroup. Similarly, in the control (CG) group, while the normal TG participants tend to show values 6% below the median, the high TG participants showed values 54% above the median, a 48% difference in in vivo vascular phenotype, with CR-PWV, P-DBP, HR, and IAD values being greater in the high TG subgroup. Interestingly, SBP (both central and peripheral) did not differ between high and normal TG subgroups, while IMT tended to be higher in the normal TG than it was in the high TG subgroup, in this CG sample.

However, in the clinical population, the high versus low TG vascular profile story somewhat differed. In the ARG group, while the normal TG participants tend to show values 48% above the median, the high TG participants showed similarly high values at 41% above the median, that is, only a 2% difference in in vivo vascular phenotype, despite the tendency for the high TG subgroup to exhibit higher P-DBP, HR, and IAD than the normal TG group in this ARG sample. Similarly, in the DAG subgroup, both the normal TG and the high TG participants showed 50% above median data, that is, no overall difference in in vivo vascular phenotype, despite the tendency for the high TG subgroup to exhibit higher IMT and P-SBP than the normal TG group within this DAG sample.

### 3.3. Between Clinical Groups Comparison of Slopes

**Figure 4:**
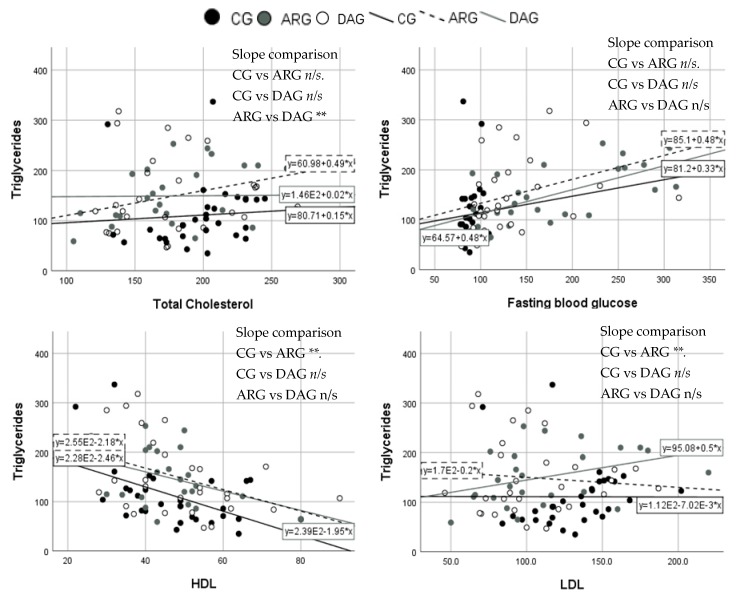
X–Y scatter plots of triglycerides (TG) (mg/dL) and the significantly correlated study markers between the clinical groups. For slope comparisons, * *p* < 0.05, ** *p* < 0.01, and n/s for non-significant difference in slopes. HDL—high-density lipoprotein; LDL—low-density lipoprotein. Please note that the scientific numbering format in the regression equations is such that for example, E2 is 10^2^. Thus, as an example, for the equation written y = 2.39E-1.95*x this corresponds to y = −1.95x + 2.39 × 10^2^.

As correlation exist between TG and some blood markers, Student’s *t*-test comparisons of the slopes between the clinical groups were run to compare the markers of interest. The increment of TG with TC was significantly greater in the DAG than in the ARG (*p =* 0.04). When comparing TG with HDL concentrations, the slope was significantly different between the CG and ARG (*p =* 0.004). The slope was also significantly greater in the CG compared with the ARG (*p =* 0.005) when comparing TG with LDL concentrations. No other slope comparison was statistically significantly. Figure 4 illustrates scatter plots and slopes of the comparisons.

### 3.4. Further Considerations

With both pooled sample triglycerides and fasting serum glucose levels not being parametric data, a spearman’s rho correlation revealed a significant association between the two variables (rho = 0.492, *p* < 0.001). Moreover, a Kruskal–Wallis comparing fasting blood sugar levels revealed ARG > DAG > CG, *p* < 0.001; with post hoc Mann–Whitney tests showing this main effect to be owing to the difference between CG and ARG, CG and DAG, as well as DAG and ARG (*p* < 0.01).

## 4. Discussion

The current study investigated key factors that influence cardio-metabolic health parameters as well as any association between these and triglycerides status in adults living in Saudi Arabia. Participants were 48.21 ± 8.3 years old with BMI of 31.9 ± 6 kg/m^2^, which is considered overweight. There was a (non-significant) tendency for BMI to increase with the complexity of health condition of the participants CG (30.6 ± 6.9), ARG (31.8 ± 4.8), and DAG (33.6 ± 5.9) kg/m^2^. Similarly, the participants’ waist to hip ratio, CG (0.86 ± 0.08 cm), ARG (0.95 ± 0.07 cm), and DAG (0.94 ± 0.13 cm), which is an indication of greater concentrations of abdominal fat, also tended to increase with complexity of the health condition. High abdominal fat is a sign of metabolic syndrome and high triglycerides levels [20] and, indeed, this association was observed in the current study (R = 0.317, *p* < 0.01).

In the current study, 82.1% of the participants had normal triglycerides concentrations, whereas 17.9% had high concentrations. TG concentration was significantly different between the clinical groups. The CG had significantly lower TG levels than the ARG (*p* < 0.01) and the DAG (*p =* 0.05). This observation would tend to indicate an association between cardiovascular health status and triglycerides levels. In addition, there was a significant difference in TG concentration between the users of statin and non-users (*p* < 0.001), so much so that when considering only non-statin users, 79.7% of the participants had normal TG concentration, whereas 20.3% had high concentration. Our observations thus illustrate the considerable impact of the usage of statin in controlling hyperlipidemia [6].

Triglycerides levels were associated with many of the blood markers of atherosclerosis in the pooled participants sample. As expected, TG was positively associated with CRP (*p* < 0.05), which is an inflammatory marker of the disease [25]. Additionally, TG was associated with higher blood glucose levels (*p* < 0.0001), which is a marker of diabetes and metabolic syndrome, and hence cardio-metabolic insufficiency [26,27]. Lastly, as expected, TG was negatively associated with HDL (*p* < 0.0001), which is a marker of hyperlipidemia [20]. These results were expected, as usually when elevated concentrations of TG occur, the rest of the lipids chain is skewed toward increase, except HDL, which decreases in these circumstances [20,28].

On the other hand, in sub-clinical groups, associations were observed between triglycerides and other blood markers of atherosclerosis. In the CG, the association was positive between the lipid profile and LDL (*p* < 0.01), while it was negatively correlated with HDL (*p* < 0.01). In the ARG, the association was positively related to FBG (*p* < 0.001), TC (*p* < 0.01), and LDL (*p =* 0.05), and negatively related to HDL (*p* < 0.05). Finally, in the DAG, the association observed was positively related to blood glucose (*p* < 0.01) and negatively related to HDL (*p* < 0.01). All of these findings support previously reported associations between TG and the blood markers of atherosclerosis [6]. These associations were expected even in the sub-clinical groups. It was also observed that elevated blood glucose levels were associated with triglyceride levels only in the unhealthy groups (ARG and DAG), which are the groups that had the highest waist to hip ratios, which are also associated with metabolic syndrome.

Further analyses indicate that the relationship slope between triglycerides and total cholesterol levels was significantly different between the ARG and DAG. The participants who were at risk of developing the disease showed a clear positive association between TG and total cholesterol, unlike the DAG, who had no significant change in the correlation. This result was not attributable to the use of statin, as the same number of participants in these two groups were using the statins. On the other hand, the slope comparison between triglycerides and LDL was significantly different between the CG and ARG. The slope in the CG shows almost no change in the relation between TG and LDL, but in the ARG, an unexpected slight negative slope has been observed. Additionally, a significant slope comparison was observed between TG and HDL. The CG group showed a lower slope than the ARG, which is expected with the elevated levels of TG.

When looking at the association between TG and the vascular structure and kinetic data in the pooled sample, many significant associations have been observed in our dataset. TG was positively associated with all of the blood pressure measurements; namely, central systolic (*p* < 0.05), peripheral systolic (*p* < 0.05), and peripheral diastolic (*p* < 0.01), which indicates an association between higher concentrations of TG and hypertension. This association was expected as the build-up of lipids in the arteries’ walls leads to narrowed arteries and higher blood pressure. A similar positive association was also observed with HR (*p* < 0.01), which is expected as it is always associated with elevated blood pressure and lipids. Additionally, TG was positively associated with CR-PWV (*p* < 0.01), as also anticipated. PWV increases with the higher stiffness of the arteries [12]. All of these results are interconnected such that the occurrence of one results in the presence of another [20].

Similar correlations were observed within the sub-population of non-statin users, which allowed any pharmacological response to drugs to be distinguished from the physiological effects of triglycerides. Thus, correlations existed between TG and C-SBP (*p* < 0.05), P-DBP (*p* < 0.05), P-DBP (*p* < 0.01), and HR (*p* < 0.01) [6]. In addition, all seven in vivo vascular phenotypes showed a 68% difference between the low and high TG levels in this non- medicated subgroup of the pooled sample. This observation indicates the huge difference between the normal and high levels of TG in relation to the vascular markers of interest. When focusing on each clinical group, the difference between normal and high levels of TG were similarly different within the CG (a 48% difference), where the CR-PWV, P-DBP, HR, and IAD values were greater in the high TG. In other words, those in vivo markers tend to be associated with high TG levels even in currently healthy participants [20], potentially indicating that our sample of controls may not have been as healthy as we may have assumed.

However, in the ARG and DAG, the overall radar profile were different in that there was no consistency for participants with a normal triglycerides level to be systematically below the median, despite the fact that this analysis was performed in participants who were not taking statin. It should, nonetheless, be noted that participants were also taking other medications that were beyond the scope of the current study to track, and hence could not be eliminated from the analyses, and as such, may have further influenced the results of our analyses. In the ARG, a high TG value exhibited higher P-DBP, HR, and IAD. In the DAG, participants with high TG exhibited higher IMT and P-SBP. These data indicate a clinical-status specific association between the in vivo vascular phenotype and TG levels in the unhealthy participants.

Triglycerides showed associations with markers of atherosclerosis in this observational study. The associations were observed in the total sample and in subgroups of clinical group, especially with the blood markers of the disease. The vascular structure markers studied correlated in the pooled sample, but not in the sub-clinical group. The association with IMT and IAD was unclear and as such, future large-scale studies are recommended to confirm or otherwise dispute our current conclusions. It is also notable that in a recent study, inactivation of the angiopoietin-like protein 4 gene (ANGPTL4) was found to result in decreased cardiovascular and atherosclerosis diseases risk, specifically coronary artery disease and ischemic stroke [29]. Thus, while it was beyond the scope of our current study to test this additional blood marker, future studies should test serum ANGPTL4 activity to assess the mechanism of triglycerides as a marker of atherosclerosis.

Finally, yet importantly, given the differences in clinical status between the three groups, it was probable that other drug use may modulate the impact of triglycerides levels on atherosclerosis disease status. In an attempt to determine if the impact of fasting blood sugar (which was clearly different between the three groups) could be controlled for, we excluded all study participants currently using a diabetes drug. However, the remaining 49 participants included 30 CG, 16 ARG, and 3 DAG participants. This thus made it clear that our study was underpowered for further data mining, owing to group size imbalance. Future studies should attempt to match blood sugar levels in the study participants groups to further elucidate the link between triglycerides and cardiovascular health.

## 5. Conclusions

Atherosclerosis is one of the major causes of morbidity worldwide and research is showing that hyperlipidemia is one of the major risk factors for developing the disease. This study pioneered observation of the impact of triglycerides on a relatively extensive list of markers of atherosclerosis. The study observed associations between triglycerides and WHR, CRP, FBG, HDL, C-SBP, P-SBP, P-DBP, CR-PWV, and HR, especially in a population ranging from healthy through to clinically diagnosed patients. Our data indicate that TG levels were associated with higher abdominal fat, diabetes, hyperlipidemia, and hypertension. Additional associations were observed in subgroups of clinical groups, even after accounting for statin use.

## Figures and Tables

**Table 1:** General characteristics of the study participants across the clinical groups. CG—control group; ARG—at-risk of developing atherosclerosis group; DAG—diagnosed with atherosclerosis group; BMI—body mass index; WHR—waist to hip ratio.

Variable	CG	ARG	DAG	Total
	*N* = 30	*N* = 25	*N* = 29	*N* = 84
**Gender**				
Male	5 (5.95%)	3 (3.57%)	6 (7.14%)	14 (16.6%)
Female	25 (29.76%)	22 (26.19%)	23 (27.38%)	70 (83.3)
**Age, years**	41.8 ± 6.8	50.12 ± 6.6	55.8 ± 6.7	48.21 ± 8.3
**BMI, kg/m^2^**	30.6 ± 6.9	31.8 ± 4.8	33.6 ± 5.9	31.9 ± 6
**WHR, cm**	0.86 ± 0.08	0.95 ± 0.07	0.94 ± 0.13	0.91 ± 0.107

Data are presented as mean ± standard deviation except gender, which is a count. In bold, are the main outcome measures.

**Table 2:** Endocrine profile and vascular structure and kinetic measurements across the clinical groups. TG—triglycerides; TC—total cholesterol; CRP—C-reactive protein; FBG—blood glucose; HDL—high-density lipoprotein; LDL—low-density lipoprotein; IQR—interquartile range; HR—heart rate; C-SBP—central systolic blood pressure; P-SBP—peripheral systolic blood pressure; P-DBP—peripheral diastolic blood pressure; CR-PWV—carotid-radial pulse wave velocity; IMT—intima-media thickness; IAD—inter-adventitial diameter.

Variable	CG	ARG	DAG	Total	*p*
	*N* = 30	*N* = 25	*N* = 29	*N* = 84	
**Use Statin**					0.001 **
Yes	0 (0%)	10 (11.9%)	10 (11.9%)	20 (23.8%)	
No	30 (35.7%)	15 (17.85%)	19 (22.6%)	64 (76.2%)	
**TG, mg/dL**					0.010 **
Median (IQR)	93 (74)	145 (93)	128 (99)	119.5 (82)	
**TC, mg/dL**					0.072
Mean ± SD	198.8 ± 32.3	182.3 ± 45.98	178.9 ± 40.59	187 ± 40.15	
**HDL, mg/dL**					0.676
Median (IQR)	48.5 (20)	43 (12)	45 (19)	45.5 (16)	
**LDL, mg/dL**					0.004 **
Mean ± SD	131.9 ± 27.65	112.4 ± 41.5	105.1 ± 34.7	116.86 ± 36.17	
**CRP, mg/dL**					0.045
Median (IQR)	0.15 (0.99)	0.50 (0.745)	0.000 (0.712)	0.41 (0.87)	
**FBG, mg/dL**					0.0001 **
Median (IQR)	90.5 (12)	162 (135)	119 (51)	104 (57)	
**P-SBP, mmHg**					0.0001 **
Median (IQR)	121 (20)	131.5 (14)	137 (23)	130.5 (18)	
**P-DBP, mmHg**					0.020 *
Mean ± SD	68.47 ± 9.86	73.9 ± 8.94	75.48 ± 10.32	72.5 ± 10.137	
**C-SBP, mmHg**					0.0001 **
Median (IQR)	106 (15)	117 (21)	123 (34)	113 (22)	
**CR-PWV, m/s**					0.071
Median (IQR)	7.1 (7.6)	10.8 (8.3)	8.9 (6.5)	9.2 (8.1)	
**HR, bpm**					0.007 **
Mean ± SD	69.5 ± 10.35	79.25 ± 11.45	73.74 ± 15.6	73.86 ± 13.17	
**IMT, mm**					0.0001 **
Median (IQR)	0.49 (0.18)	0.556 (0.19)	0.696 (0.35)	0.575 (0.25)	
**IAD, mm**					0.004 **
Mean ± SD	6.32 ± 0.62	6.61 ± 0.68	7.03 ± 0.94	6.65 ± 0.81	

Data are presented as mean ± standard deviation for normally distributed data, and median (interquartile range) for non normally distributed sample, except for the use of statin, which is presented as count and percentage (%) of the total. Significant differences between the clinical groups are indicated by (* = *p* < 0.05) and (** = *p* < 0.01). In bold are the names of the outcome variables of interest.

## References

[1] BHF Atherosclerosis. https://www.bhf.org.uk/heart-health/conditions/atherosclerosis.

[2] Libby P. (2006). Atherosclerosis: Disease biology affecting the coronary vasculature. Am. J. Cardiol..

[3] Adams A., Bojara W., Schunk K. (2017). Early diagnosis and treatment of coronary heart disease in symptomatic subjects with advanced vascular atherosclerosis of the carotid artery (type III and IV b findings using ultrasound). Cardiol. Res..

[4] Lertratanakul A., Bernatsky S., Hanly J.G., Isenberg D., Rahman A., Merrill J., Wallace D.J., Ginzler E., Khamashta M., Bruce I. (2014). 25-hydroxyvitamin D and cardiovascular disease in patients with systemic lupus erythematosus: Data from a large international inception cohort. Arthritis Care Res..

[5] Sibley C.T., Estwick T., Zavodni A., Huang C.Y., Kwan A.C., Soule B.P., Long Priel D.A., Remaley A.T., Rudman Spergel A.K., Turkbey E.B. (2014). Assessment of atherosclerosis in chronic granulomatous disease. Circulation.

[6] Nordestgaard B.G., Varbo A. (2014). Triglycerides and cardiovascular disease. Lancet.

[7] George S.J., Johnson J. (2010). Atherosclerosis: Molecular and Cellular Mechanisms.

[8] Inaba Y., Chen J.A., Bergmann S.R. (2010). Prediction of future cardiovascular outcomes by flow-mediated vasodilatation of brachial artery: A meta-analysis. Int. J. Cardiovasc. Imaging.

[9] Libby P., Ridker P.M. (2004). Inflammation and atherosclerosis: Role of c-reactive protein in risk assessment. Am. J. Med..

[10] URMC (2018). Atherosclerosis. Health Encyclopedia.

[11] Rubinshtein R., Kuvin J.T., Soffler M., Lennon R.J., Lavi S., Nelson R.E., Pumper G.M., Lerman L.O., Lerman A. (2010). Assessment of endothelial function by non-invasive peripheral arterial tonometry predicts late cardiovascular adverse events. Eur. Heart J..

[12] McEniery C.M., Wallace S., Mackenzie I.S., McDonnell B., Yasmin, Newby D.E., Cockcroft J.R., Wilkinson I.B. (2006). Endothelial function is associated with pulse pressure, pulse wave velocity, and augmentation index in healthy humans. Hypertension.

[13] Kim J.S., Caplan L.R., Wong K.S.L. (2008). Intracranial Atherosclerosis.

[14] Polak J.F., Szklo M., Kronmal R.A., Burke G.L., Shea S., Zavodni A.E., O’Leary D.H. (2013). The value of carotid artery plaque and intima-media thickness for incident cardiovascular disease: The multi-ethnic study of atherosclerosis. J. Am. Heart Assoc..

[15] Awad I.A., Abbas H.Y. (2017). Ultrasound evaluation of carotid artery intima-media thickness in patients with risk factors for cardiovascular disease. Int. J. Diagn. Imaging.

[16] Arora T., Rehan H.S. (2015). A perspective on role of calcium and vitamin d in cardiovascular outcomes and lipid profile. J. Basic Clin. Physiol. Pharmacol..

[17] Hajj A., Chedid R., Chouery E., Megarbane A., Gannage-Yared M.H. (2016). Relationship between vitamin D receptor gene polymorphisms, cardiovascular risk factors and adiponectin in a healthy young population. Pharmacogenomics.

[18] Zheng C., Khoo C., Furtado J., Sacks F.M. (2010). Apolipoprotein C-III and the metabolic basis for hypertriglyceridemia and the dense low-density lipoprotein phenotype. Circulation.

[19] Brown W.V. (2007). High-density lipoprotein and transport of cholesterol and triglyceride in blood. J. Clin. Lipidol..

[20] Talayero B.G., Sacks F.M. (2011). The role of triglycerides in atherosclerosis. Curr. Cardiol. Rep..

[21] Berdanier C.D., Zempleni J. (2009). Advanced Nutrition: Macronutrients, Micronutrients, and Metabolism.

[22] Lilley J.S., Linton M.F., Kelley J.C., Graham T.B., Fazio S., Tavori H. (2017). A case of severe acquired hypertriglyceridemia in a 7-year-old girl. J. Clin. Lipidol..

[23] Ikonomidis I., Ntai K., Kadoglou N., Papadakis I., Kornelakis M., Tritakis V., Varoudi M., Papadima T., Triantafyllidi H., Parissis J. (2013). The evaluation of pulse wave velocity using arteriograph and complior apparatus across multiple cohorts of cardiovascular-related diseases. Int. J. Cardiol..

[24] Roman M.J., Naqvi T.Z., Gardin J.M., Gerhard-Herman M., Jaff M., Mohler E. (2006). Clinical application of noninvasive vascular ultrasound in cardiovascular risk stratification. Vasc. Med..

[25] van Wissen S., Trip M.D., Smilde T.J., de Graaf J., Stalenhoef A.F., Kastelein J.J. (2002). Differential HS-CRP reduction in patients with familial hypercholesterolemia treated with aggressive or conventional statin therapy. Atherosclerosis.

[26] Hamburg N.M., Keyes M.J., Larson M.G., Vasan R.S., Schnabel R., Pryde M.M., Mitchell G.F., Sheffy J., Vita J.A., Benjamin E.J. (2008). Cross-sectional relations of digital vascular function to cardiovascular risk factors in the framingham heart study. Circulation.

[27] Beckman J.A., Creager M.A., Libby P. (2002). Diabetes and atherosclerosis: Epidemiology, pathophysiology, and management. JAMA.

[28] Chapman M.J., Ginsberg H.N., Amarenco P., Andreotti F., Borén J., Catapano A.L., Descamps O.S., Fisher E., Kovanen P.T., Kuivenhoven J.A. (2011). Triglyceride-rich lipoproteins and high-density lipoprotein cholesterol in patients at high risk of cardiovascular disease: Evidence and guidance for management. Eur. Heart J..

[29] Yang Q., Yin R.-X., Cao X.-L., Huang F., Zhou Y.-J., Chen W.-X. (2018). Angptl4 variants and their haplotypes are associated with serum lipid levels, the risk of coronary artery disease and ischemic stroke and atorvastatin cholesterol-lowering responses. Nutr. Metab..

